# Supplementary material for: Expanded Performance Comparison of the Oncuria 10-Plex Bladder Cancer Urine Assay Using Three Different Luminex xMAP Instruments
Source: Diagnostics (Basel). 2025 Jul 10;15(14):1749. doi: 10.3390/diagnostics15141749 (PMC12294033; doi:10.3390/diagnostics15141749)
Supplement: Supplementary file 1 [file diagnostics-15-01749-s001.zip › Supplemental Table S1--Raw Fluorescence Outputs.pdf]

Supplemental Table S1. Example of raw fluorescence data outputs across three flow analyzers (median fluorescence intensity, arbitrary units)

Sample ID	Instrument	A1AT	ANG	ApoE	CA9	IL-8	MMP-9	MMP-10	PAI-1	SDC1	VEGF-A
#M003	**FlexMap 3D**	20921	62449	15767	250	37380	5756	1351	17733	20757	29158
**Cancer**	**200**	2435	7421	1844	34	4619	702	154	2006	2333	3580
	**MagPix**	2334	8235	1770	24	4551	553	117	1906	2313	3140
#M004	**FlexMap 3D**	20411	4136	1841	61	66738	1919	6	1215	18055	3041
**Cancer**	**200**	2321	458	214	8	8592	226	0	139	2132	369
	**MagPix**	2281	457	183	7	8748	165	0	128	2003	322
#M006	**FlexMap 3D**	16548	7808	1997	16	10606	310	11	1272	23569	922
**Cancer**	**200**	1948	959	228	2	1323	36	1	151	2776	112
	**MagPix**	1884	946	201	1	1178	24	1	138	2714	92
#C017	**FlexMap 3D**	1224	578	140	9	18	111	-6	7	1984	17
**Control**	**200**	147	61	19	2	2	10	0	1	228	1
	**MagPix**	143	59	19	1	2	12	-1	1	225	2
#C018	**FlexMap 3D**	789	43	108	1	9	105	5	60	576	52
**Control**	**200**	93	3	11	2	2	10	0	7	62	5
	**MagPix**	82	4	8	0	1	12	0	4	61	6
#C019	**FlexMap 3D**	9715	1120	201	0	81	44	-9	28	2128	229
**Control**	**200**	1176	119	21	0	10	4	1	2	233	28
	**MagPix**	1214	120	23	0	8	4	0	3	241	29

Shown is output from six representative urine samples, from three confirmed BC subjects (“Cancer”) and three Control subjects. Values are averages of duplicate wells per analyte, per subject, rounded to the nearest whole number.
