# Supplementary material for: Expanded Performance Comparison of the Oncuria 10-Plex Bladder Cancer Urine Assay Using Three Different Luminex xMAP Instruments
Source: Diagnostics (Basel). 2025 Jul 10;15(14):1749. doi: 10.3390/diagnostics15141749 (PMC12294033; doi:10.3390/diagnostics15141749)
Supplement: Supplementary file 1 [file diagnostics-15-01749-s001.zip › Supplemental Table S2--Protein in Case vs Control 26JUN2025.pdf]

Supplemental Table S2. Oncuria analyte levels (pg/mL) in BC Case versus Control urine specimens

		All Samples						BC Cases						Controls						
**Instrument**	**Analyte**	**N**	**mean**	**sd**	**min**	**median**	**max**	**n**	**mean**	**sd**	**min**	**median**	**max**	**n**	**mean**	**sd**	**min**	**median**	**max**	**P**
FlexMap 3D	MMP9	181	397	1,701	20	20	14,680	54	527	2,009	20	82	14,680	127	342	1,558	20	20	14,680	.55
FlexMap 3D	IL8	181	170	373	2	9	1,410	54	358	486	2	67	1,410	127	90	279	2	3	1,410	.0003
FlexMap 3D	VEGFA	181	165	499	12	43	4,836	54	395	865	12	98	4,836	127	67	91	12	26	570	.007
FlexMap 3D	CA9	181	7	26	2	2	287	54	15	47	2	2	287	127	3	6	2	2	47	.08
FlexMap 3D	SDC1	181	9,540	6,848	153	8,005	36,675	54	12,092	8,306	643	10,225	36,675	127	8,454	5,830	153	7,156	26,734	.005
FlexMap 3D	PAI1	181	198	656	12	12	4,642	54	541	1,097	12	52	4,642	127	52	194	12	12	1,960	.002
FlexMap 3D	APOE	181	8,882	1,874	400	400	20,250	54	1,647	3,244	400	429	20,250	127	565	491	400	400	3,307	.02
FlexMap 3D	A1AT	181	102,710	140,888	611	31,677	445,620	54	154,514	157,593	3,685	76,145	445,620	127	80,684	127,556	611	21,998	445,620	.003
FlexMap 3D	ANG	181	472	784	5	99	3,500	54	949	1,146	5	393	3,500	127	268	432	5	61	2,481	.0001
FlexMap 3D	MMP10	181	32	176	12	12	2,310	54	67	319	12	12	2,310	127	17	25	12	12	245	.25
LX200	MMP9	181	386	1,686	20	20	14,680	54	515	2,007	20	71	14,680	127	332	1,535	20	20	14,680	.55
LX200	IL8	181	167	369	2	9	1,410	54	353	483	2	64	1,410	127	88	274	2	3	1,410	.0003
LX200	VEGFA	181	160	476	12	40	4,397	54	383	824	12	96	4,397	127	64	88	12	20	567	.006
LX200	CA9	181	7	26	2	2	285	54	15	46	2	2	285	127	3	6	2	2	49	.07
LX200	SDC1	181	9,470	6,934	153	8,209	38,813	54	12,160	8,601	580	10,277	38,813	127	8,326	5,757	153	6,947	26,065	.004
LX200	PAI1	181	198	658	12	12	4,599	54	540	1,100	12	51	4,599	127	52	193	12	12	1,956	.002
LX200	APOE	181	866	1,813	400	400	19,404	54	1,600	3,142	400	419	19,404	127	554	464	400	400	3,232	.02
LX200	A1AT	181	102,213	139,719	611	32,693	445,620	54	155,324	157,297	3,687	80,399	445,620	127	79,630	125,529	611	21,046	445,620	.002
LX200	ANG	181	468	782	5	96	3,500	54	950	1,150	5	388	3,500	127	263	420	5	59	2,330	.0001
LX200	MMP10	181	32	181	12	12	2,383	54	67	328	12	12	2,383	127	17	25	12	12	247	.26
MagPix	MMP9	181	387	1,692	20	20	14,680	54	513	2,006	20	68	14,680	127	334	1,546	20	20	14,680	.56
MagPix	IL8	181	170	373	2	10	1,410	54	358	487	2	64	1,410	127	89	278	2	3	1,410	.0003
MagPix	VEGFA	181	159	466	12	38	4,353	54	379	805	12	94	4,353	127	65	90	12	19	572	.006
MagPix	CA9	181	7	27	2	2	290	54	15	47	2	2	290	127	3	6	2	2	49	.07
MagPix	SDC1	181	9,553	7,208	153	8,241	44,261	54	12,508	9,151	621	10,257	44,261	127	8,296	5,801	153	6,874	26,885	.003
MagPix	PAI1	181	196	658	12	12	4,756	54	540	1,102	12	49	4,756	127	50	189	12	12	1,936	.002
MagPix	APOE	181	829	1,676	400	400	17,735	54	1,521	2,905	400	400	17,735	127	535	416	400	400	2,661	.02
MagPix	A1AT	181	101,740	138,853	611	31,503	445,620	54	156,376	155,892	3,695	90,026	445,620	127	78,508	124,489	611	19,665	445,620	.002
MagPix	ANG	181	472	781	5	98	3,500	54	958	1,141	5	432	3,500	127	266	425	5	59	2,347	.0001
MagPix	MMP10	181	31	178	12	12	2,355	54	66	323	12	12	2,355	127	17	24	12	12	229	.26
