# Supplementary figures and images for: Expanded Performance Comparison of the Oncuria 10-Plex Bladder Cancer Urine Assay Using Three Different Luminex xMAP Instruments
Source: Diagnostics (Basel). 2025 Jul 10;15(14):1749. doi: 10.3390/diagnostics15141749 (PMC12294033; doi:10.3390/diagnostics15141749)

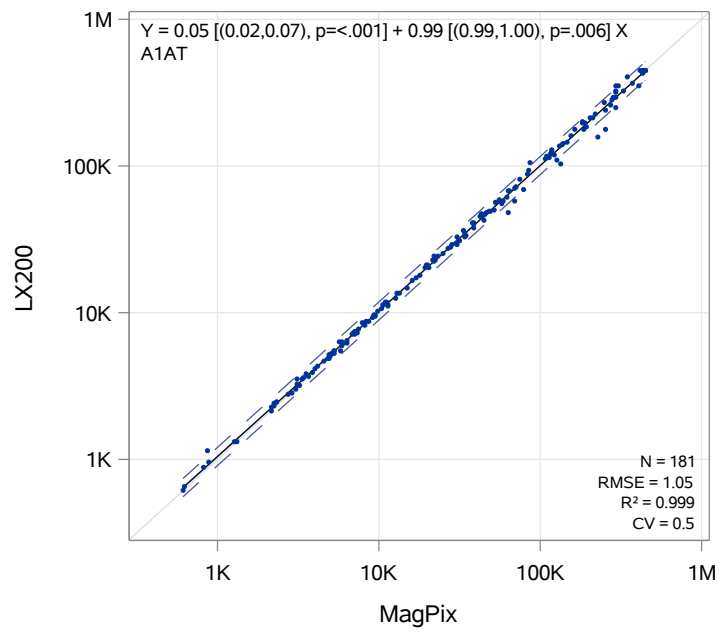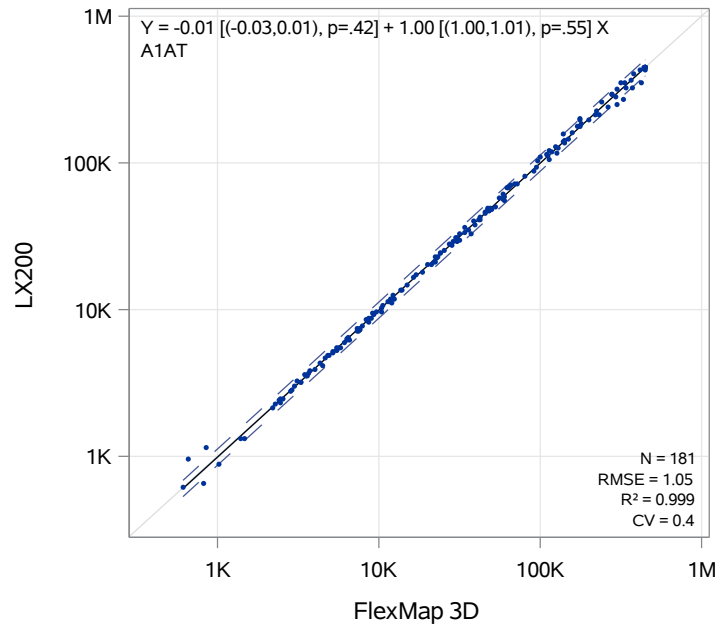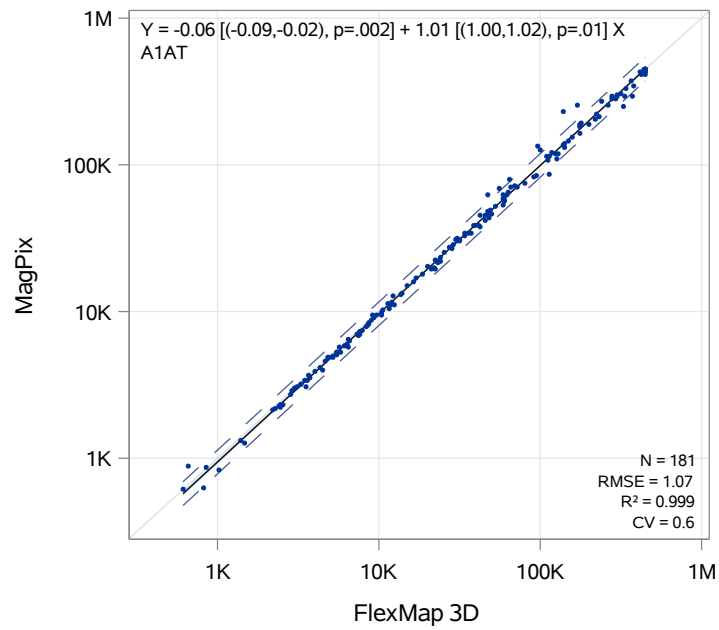

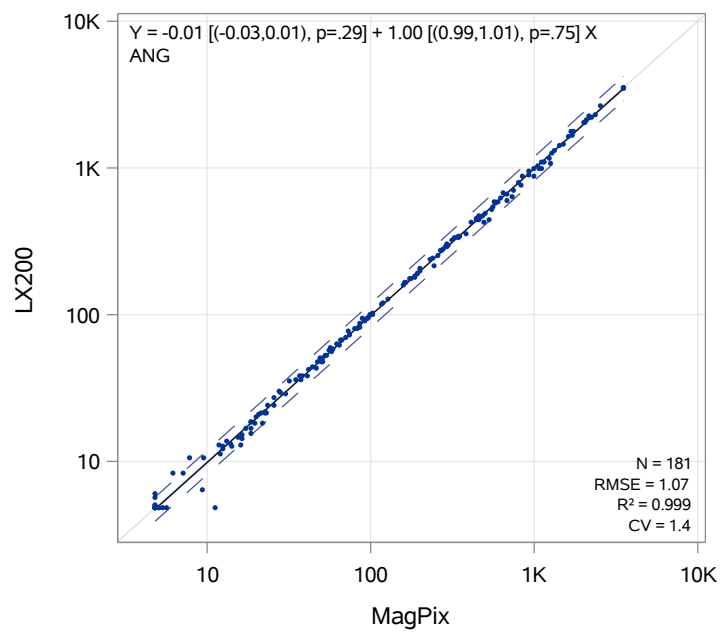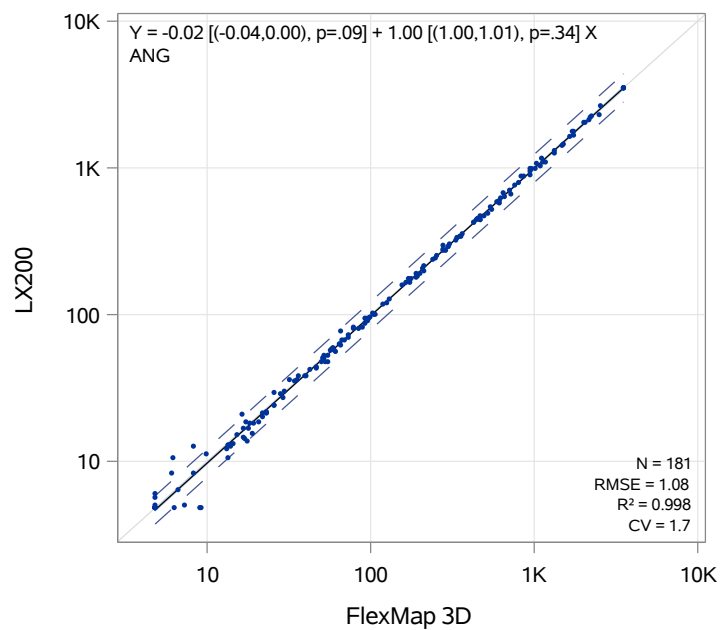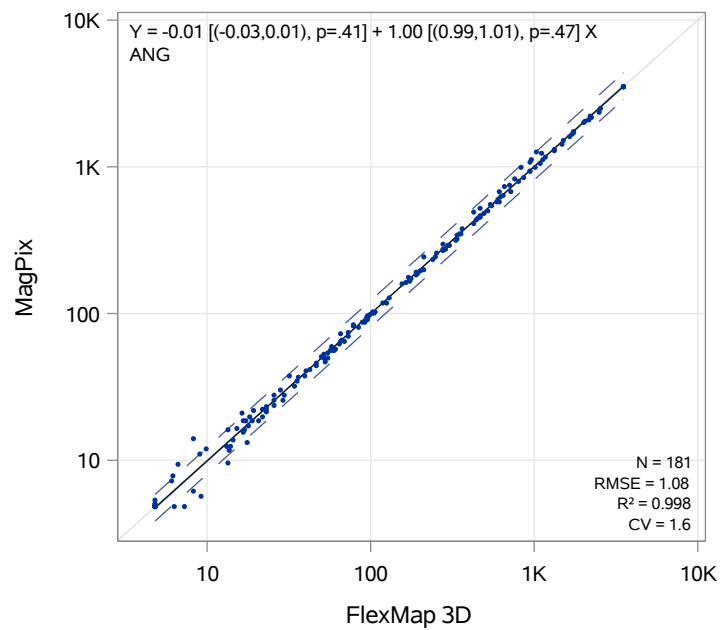

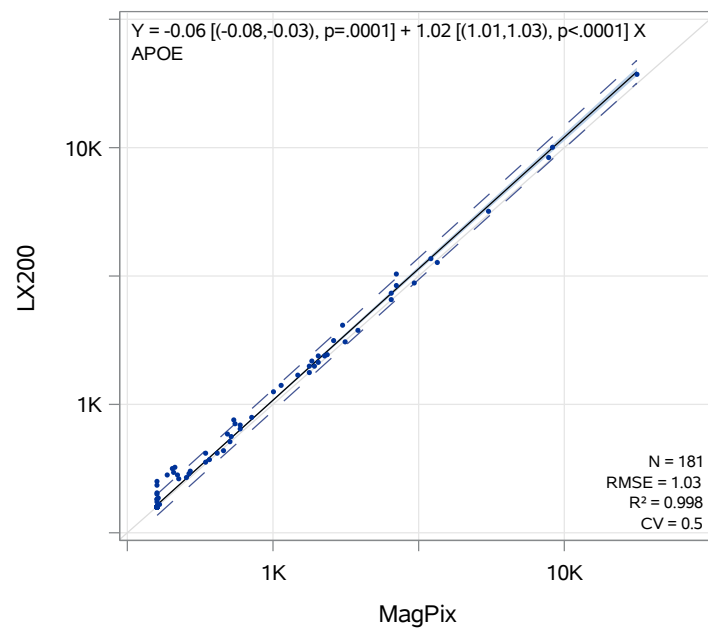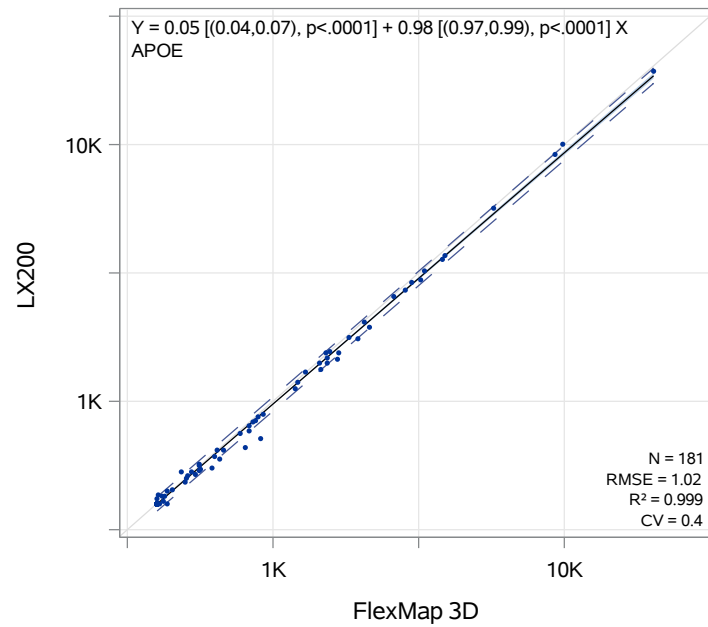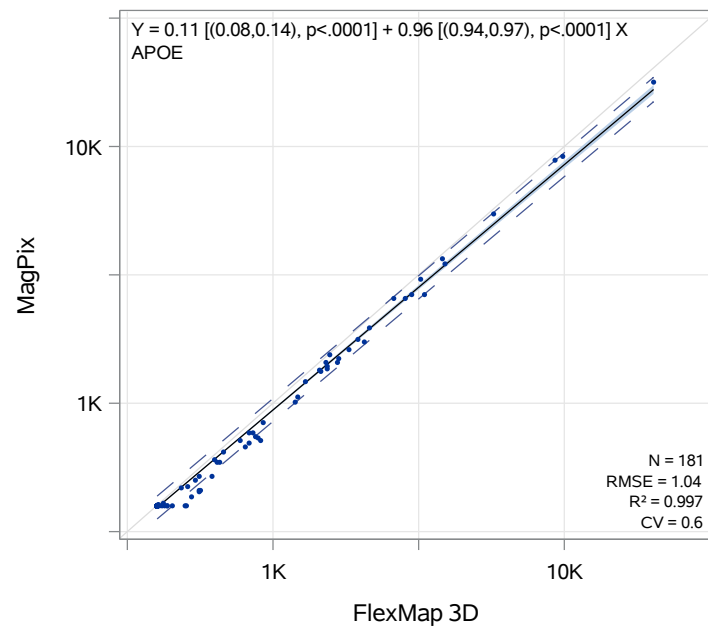

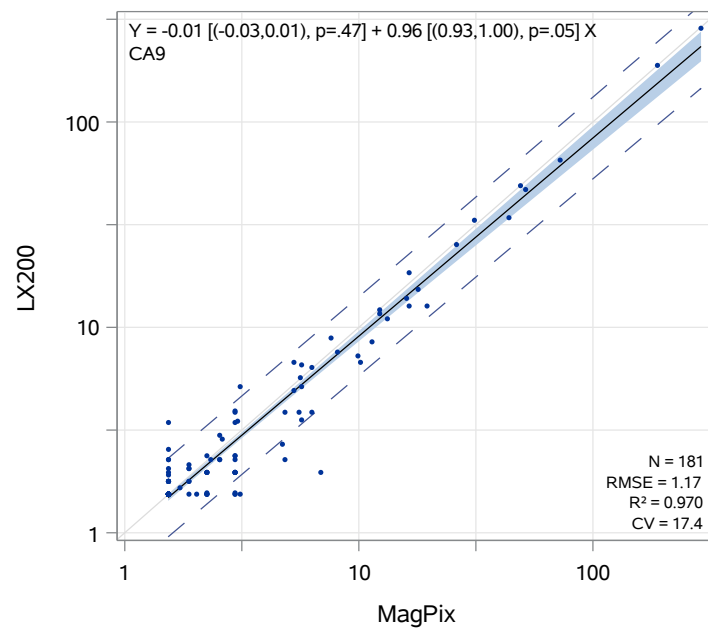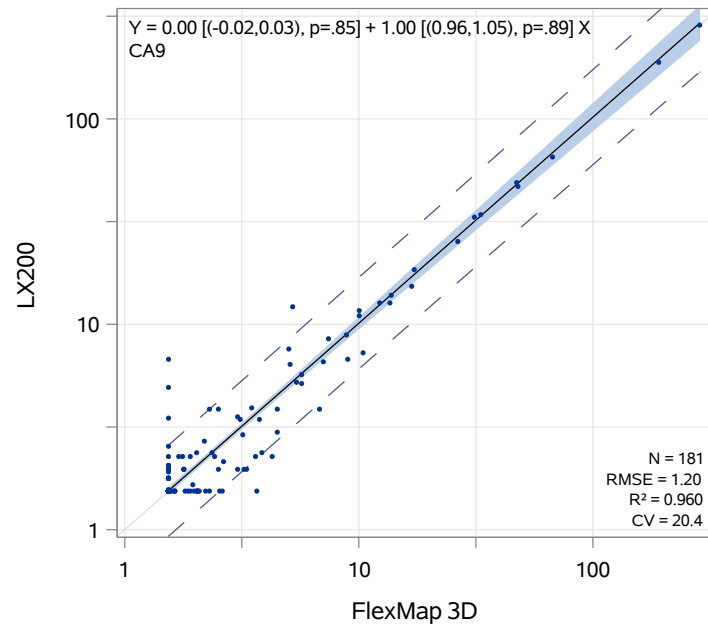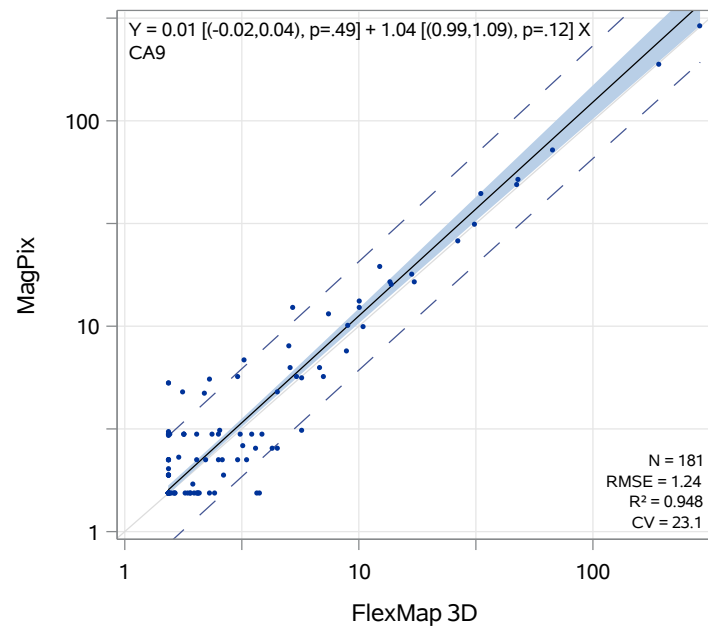

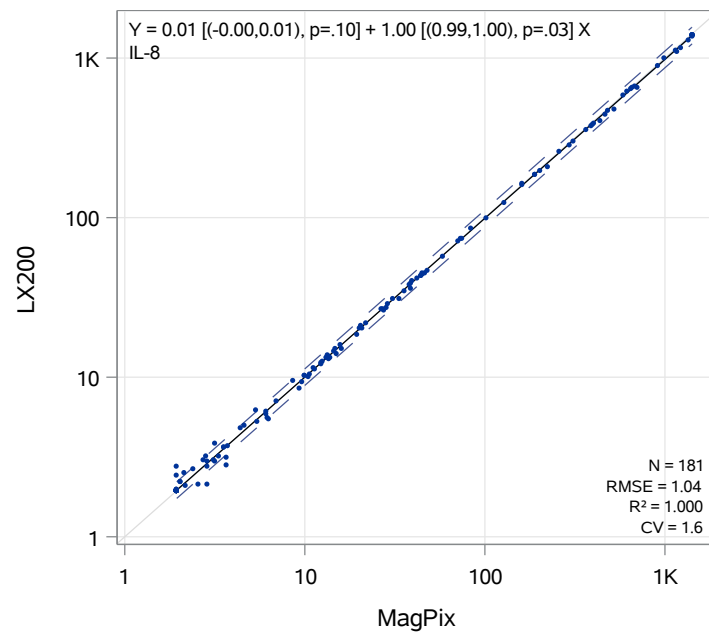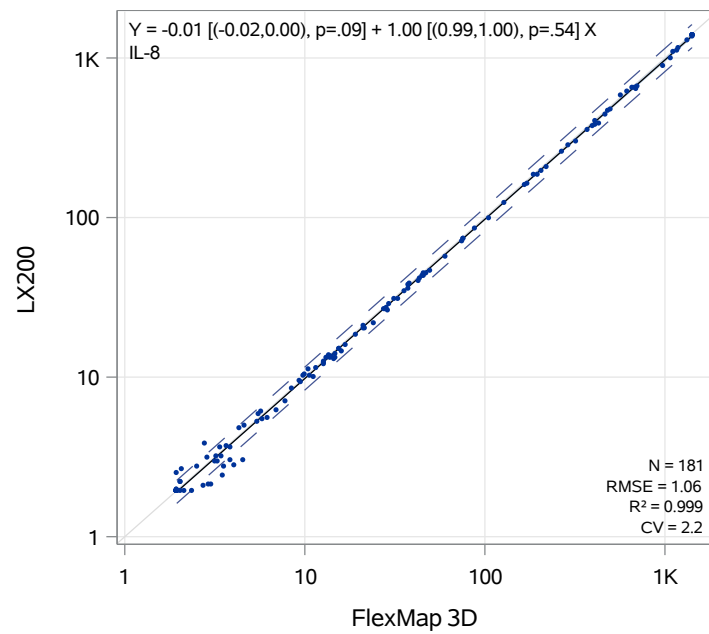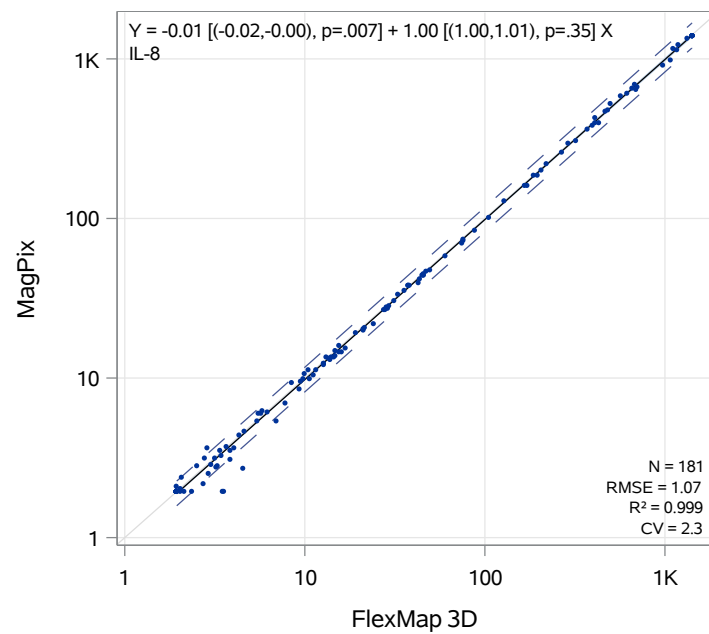

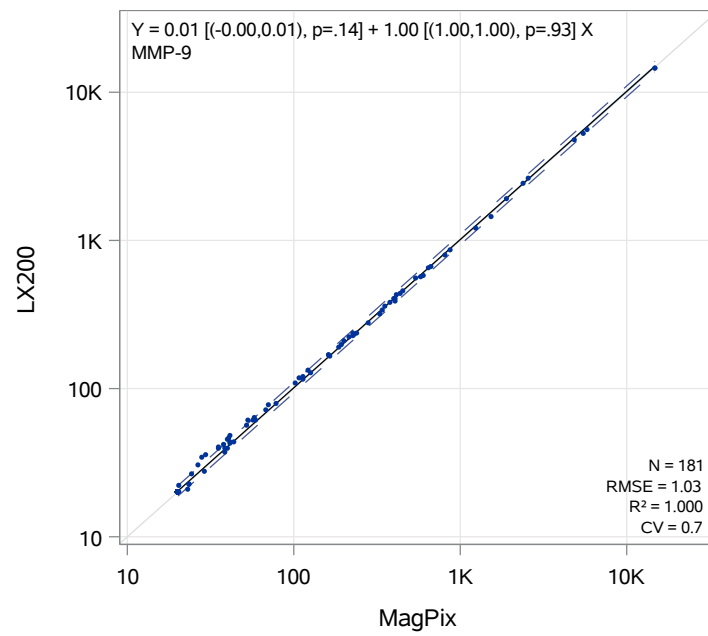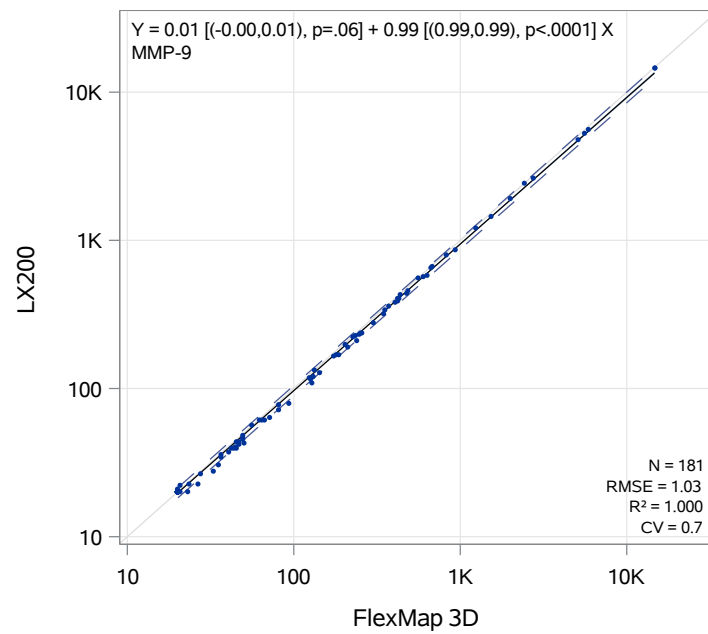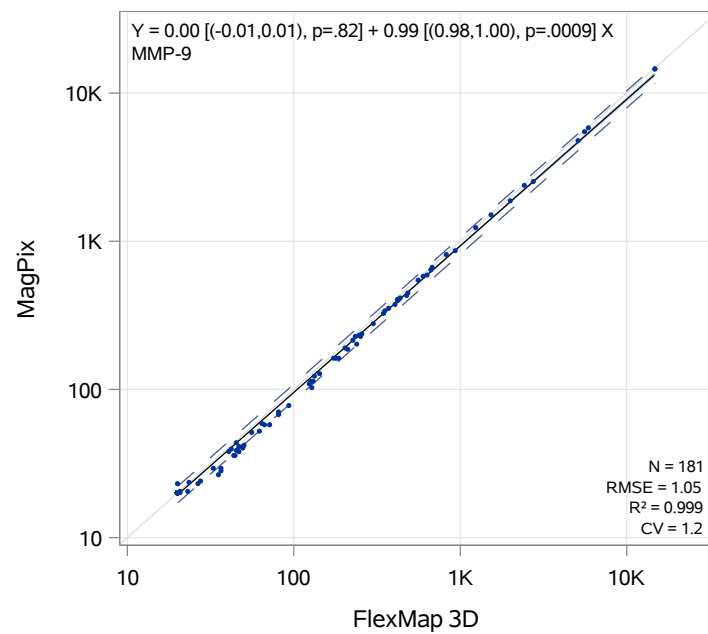

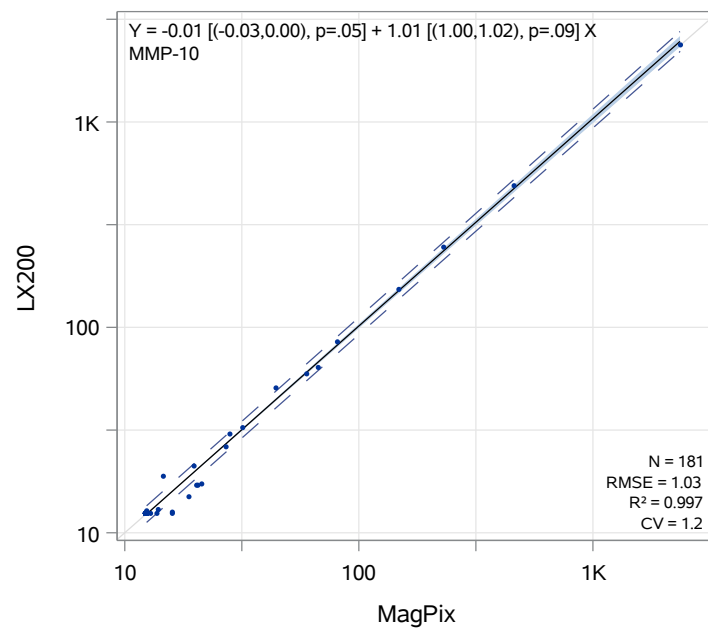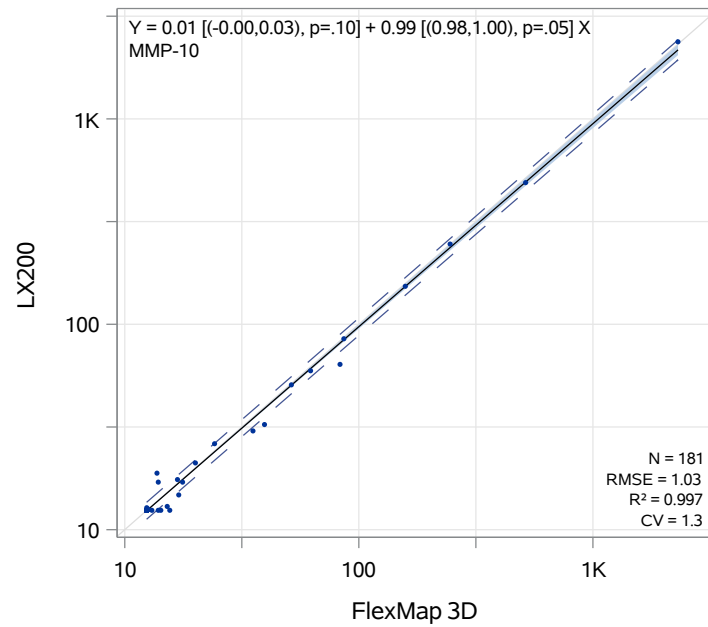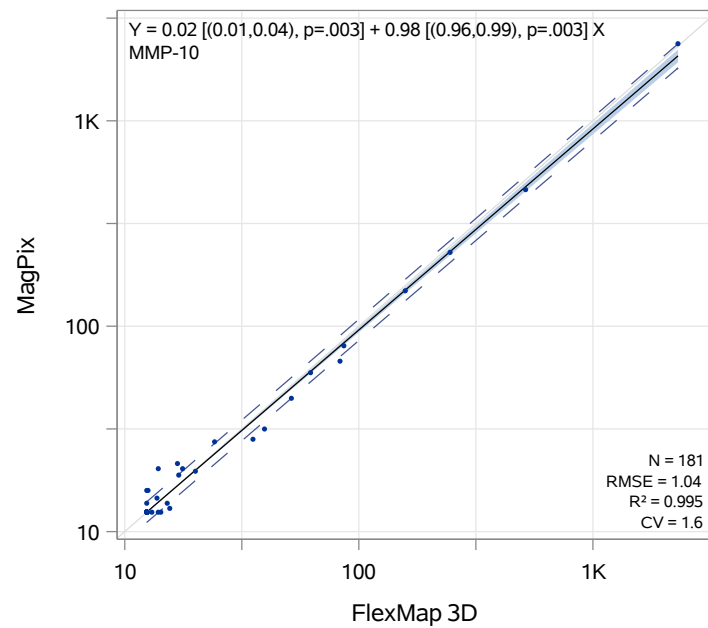

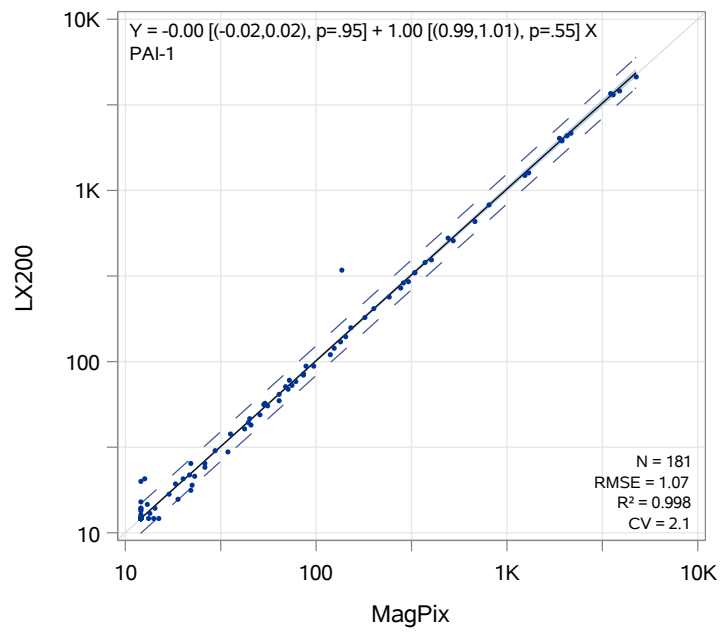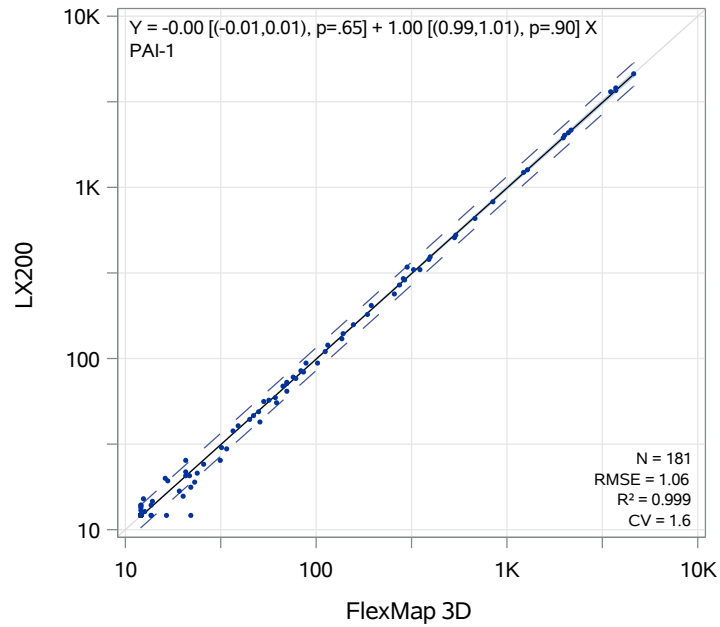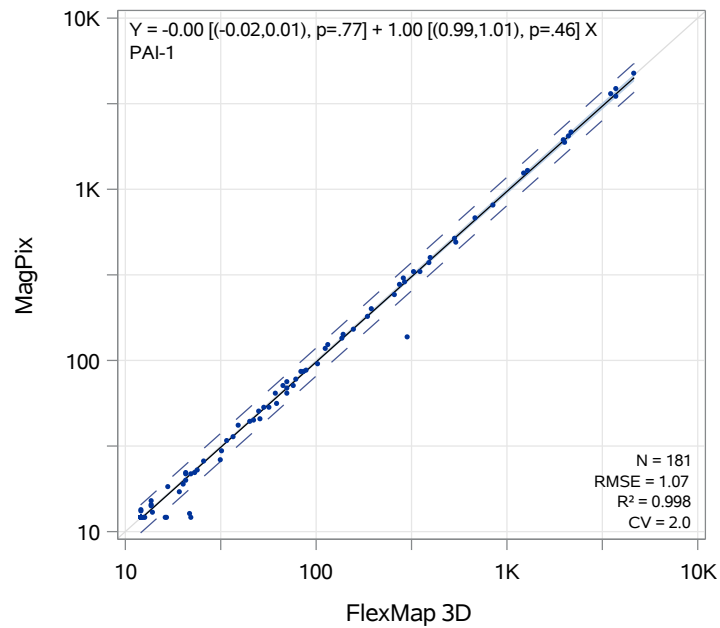

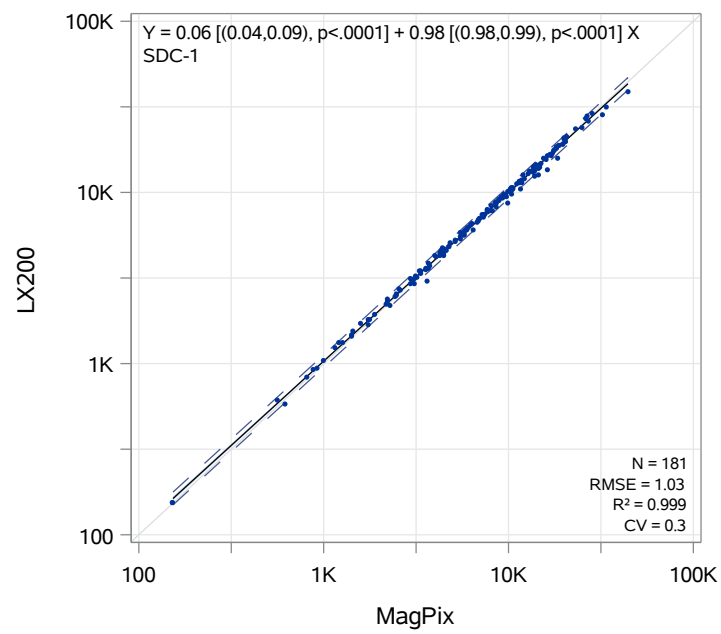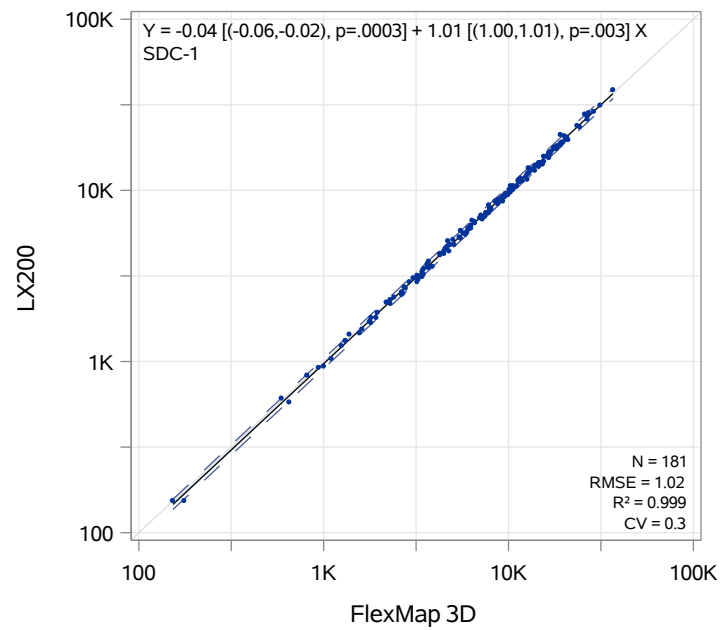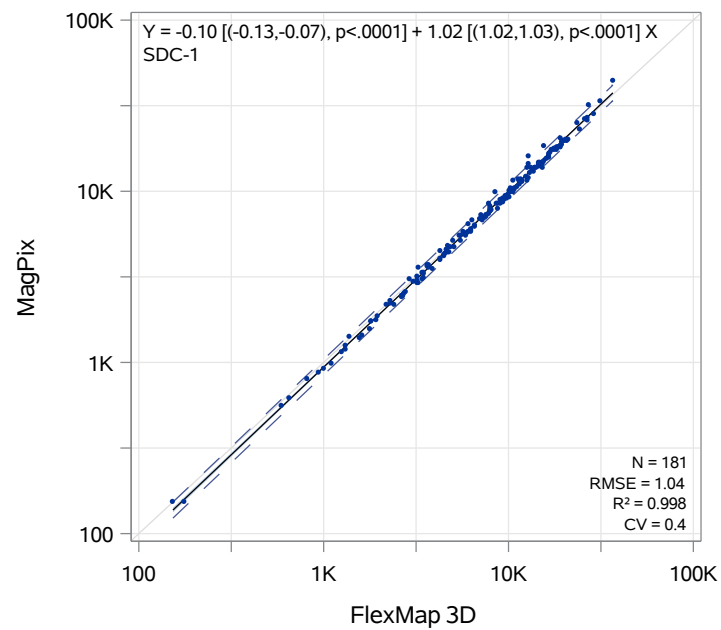

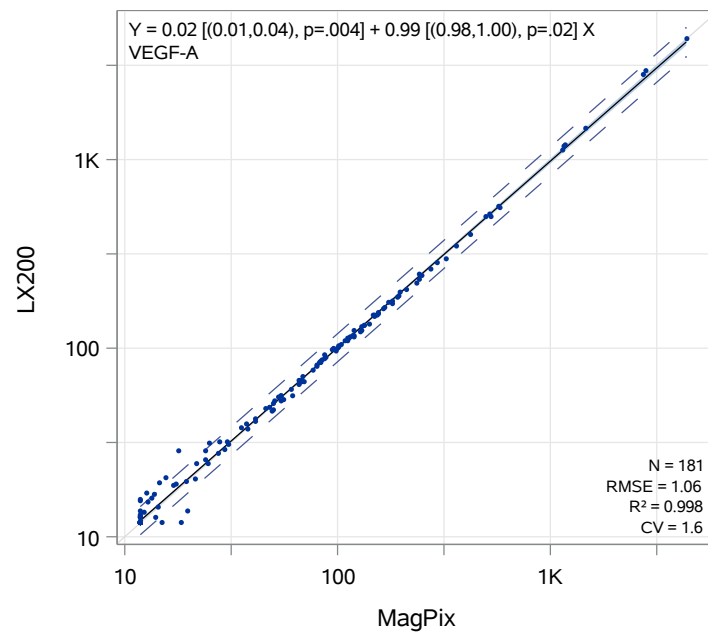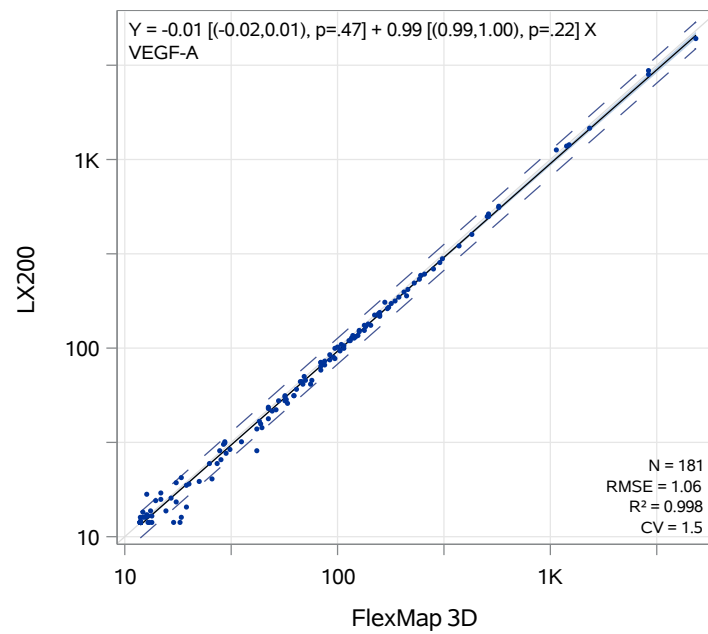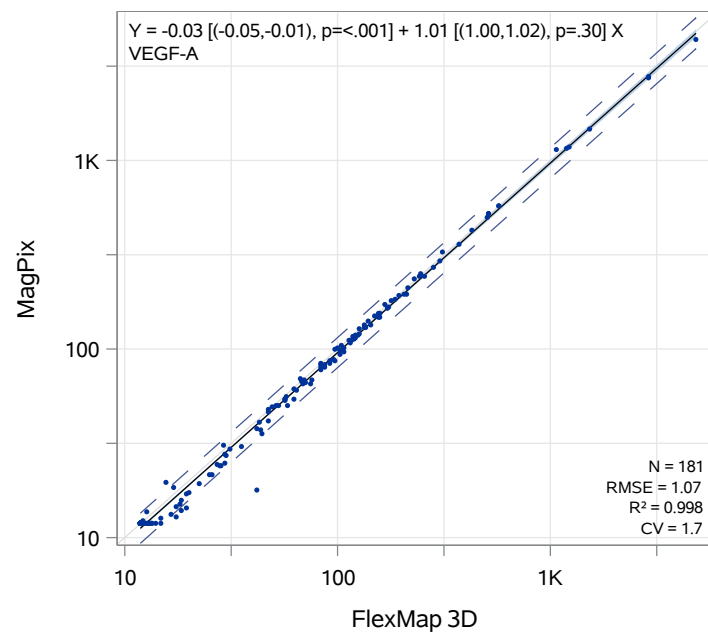

Supplement: Supplementary file 1 [file diagnostics-15-01749-s001.zip › Supplemental Figure S1--Pairwise Correlation Plots.pdf]
